# The Effect of Increasing Tibial Tuberosity Advancement and Quadriceps Muscle Force on Cranial Translation of the Tibia in the Cranial Cruciate Deficient Stifle Joint in Dogs

**DOI:** 10.3389/fvets.2022.914763

**Published:** 2022-06-30

**Authors:** Ciprian Ober, Chen Berger, Liat Cohen, Joshua Milgram

**Affiliations:** ^1^Department of Surgery and Intensive Care, Faculty of Veterinary Medicine, University of Agricultural Sciences and Veterinary Medicine, Cluj-Napoca, Romania; ^2^The Laboratory of Biomechanics, Koret School of Veterinary Medicine, The Robert H. Smith Faculty of Agriculture, Food and Environment, Hebrew University of Jerusalem, Jerusalem, Israel

**Keywords:** tibial tuberosity advancement, cruciate ligament, quadriceps, *in-vitro*, canine, quadriceps muscle

## Abstract

**Background:**

Cranial cruciate ligament (CCL) disease is a well-known pathology that generates both rotational and translational instability of the stifle joint that leads to osteoarthritis in dogs. Tibial tuberosity advancement (TTA) is a common surgical technique used to dynamically neutralize the tibiofemoral shear forces to achieve stifle joint stability. However, significant persistent instability has been documented in clinical cases. The purpose of this study was to evaluate the effect of increasing quadriceps load, increasing tibial tuberosity advancement, and increasing joint flexion angle on the cranial translation of the tibia relative to the femur in the cranial cruciate ligament deficient stifle joint.

**Methods and Results:**

Six cadaveric hind limbs were collected from six healthy mixed breed dogs of medium build and prepared for biomechanical testing. The specimen was placed into a custom-made joint testing machine, and translation of the tibia relative to the femur was measured at stifle angles of 135°, 120°, and 105°. Cranial tibial thrust was generated by applying a vertical load to the metatarsal pad and the quadriceps muscle was simulated with loads of 0, 5, and 10 kg applied to the patella *via* a system of weights and pulleys. All specimens were tested with the CCL intact and cut, both of which served as controls. The tibial tuberosity was then advanced using both 6 mm and 9 mm cages, and the specimen was tested using the identical technique. Each specimen was loaded to failure by increasing the load applied to the pes until the sudden marked cranial translation of the tibia. Tibial tuberosity advancement with an applied quadriceps load was successful in limiting cranial tibial translation in 54/62 tests. Under similar loading conditions, TTA failed to limit cranial translation in 8 tests. The failures occurred at all angles of flexion examined. In the cases that failed cranial translation could be limited by either increasing the quadriceps load or increasing the amount of tibial tuberosity advancement.

**Conclusion:**

This study showed that TTA with an applied quadriceps load is effective at decreasing cranial tibial translation at functional joint angles.

## Introduction

Tibial tuberosity advancement (TTA) is a technique for the dynamic stabilization of the CCL-deficient stifle joint (1–4). When performing a TTA the insertion of the quadriceps muscle is moved cranially by performing an osteotomy of the tibial tuberosity in the frontal plane, and moving it cranially until the patella tendon angle (PTA), with the hind leg in extension, is 90° (5). Clinical outcome is, in general, excellent; however, it has been shown that regardless of the technique performed, the operated limb does not regain full function, and significant instability persists in treated cases (6–8).

The rationale behind the TTA is the initial assumption that the direction of the resultant joint force is approximately parallel to the patella tendon. When the patella tendon force is resolved into its two component forces with the stifle positioned such that the PTA > 90°, the horizontal component of the force is cranially directed and parallel to the joint surface, resulting in tibio-femoral shear. The direction of the tibio-femoral shear force is reversed when the PTA is < 90°. When a TTA is performed, according to current recommendations, the PTA with the stifle in extension is decreased to an angle of 90°. With a PTA of 90°, the horizontal component of the force in the patella tendon is zero, resulting in a single component, parallel to the resultant joint force. As the joint is flexed during the stance phase, the PTA decreases, resulting in a caudally directed tibio-femoral shear force of increasing magnitude, with expected increased stability of the joint (3–5, 9).

Several *in vitro* biomechanical studies have been published which have examined the effect of TTA on the biomechanics of the canine stifle (10–12). Although these studies have validated the ability of TTA to stabilize the CCL deficient stifle joint, questions still remain as to the effect of joint angle, quadriceps load, as well as the extent to which the tibial tuberosity should be advanced. In this study, the objective was to evaluate the effect of increasing quadriceps load, increasing tibial tuberosity advancement, and increasing joint flexion angle on the cranial translation of the tibia relative to the femur in the CCL deficient stifle joint.

## Materials and Methods

### Samples

Six stifle joints were harvested from six skeletally mature dogs weighing between 20 and 25 kg by dislocation of the coxofemoral joint. The specimens were obtained from dogs free of locomotor deficits and euthanized for reasons unrelated to this study and donated to this study with client consent. The stifle joints were radiographed in two orthogonal views and were only included in this study if they were found to be skeletally mature, free of radiographic evidence of degenerative joint disease, and had a TPA of 24° ± 1°. Plate and cage size was determined on the lateral radiograph using the TTA template provided by Kyon^®^. Only specimens requiring a 5-hole plate and 6 mm of TTAwere included in this study. Once the specimen was found to be suitable for this study it was wrapped in saline soaked gauze and stored at −20°C.

### Description of Experimental Equipment

Cranial translation of the tibia was measured using the “Nest of Birds” electromagnetic tracking system (Ascension Technology, Inc., Burlington, VT). The Nest of Birds consists of an electronic unit, a transmitter, and 4 sensors with a reported accuracy of 0.5 mm. The sensors are able to measure the magnetic field generated by the transmitter while the electronic unit controls the transmitted signal. From signals measured by the sensors, the system calculates the position and orientation of the sensors within the generated magnetic field. The “Nest of Birds” was attached to a personal computer and the data was collected using software provided by the manufacturer.

The custom-built joint testing machine was manufactured entirely from non-ferromagnetic materials and was designed to rigidly hold the specimen during testing by means of a custom designed bone clamp. The bone clamp was designed so that it could be secured within the joint testing machine as well as the alignment chamber. The bone clamp could be unlocked to allow movement of the specimen and locked once the ideal position was achieved, without the need to unclamp the specimen. The location of the specimen in the joint testing machine was standardized using the alignment chamber which is a custom-built, radiolucent frame with embedded radio-opaque markers. Each specimen was radiographed in 2 orthogonal views, and the location was adjusted until bone landmarks on the femur coincided with predetermined radio-opaque markers.

### Sample Preparation

On the day of testing the specimen was thawed in a saline bath at room temperature, and stripped of all soft tissues leaving the skin and soft tissues around the stifle joint, crus, and pes intact. A transverse osteotomy of the femur at the level of the proximal metaphysis was performed 14 cm proximal to the level of the tibial plateau. The proximal femur was then potted in polymethylmethacrylate (PMMA) using a custom-made mold.

The proximal femur was then secured within a clamp which was custom designed to allow it to be bolted into both an alignment chamber and joint testing machine. The design of the clamp allowed the specimen to be removed from the joint testing machine during testing and returned to its identical location. The specimen was first bolted into the alignment chamber and radiographed in two orthogonal views. The location of the specimen was altered by unlocking the clamp, and adjusting it until it was aligned with designated radio-opaque markers in both the mediolateral and craniocaudal radiographic views. Once the ideal position had been achieved the clamp was locked which prevented relative movement between the femur and the clamp.

Once aligned, a single sensor was rigidly attached to the tibia using a 5 mm wooden dowel and the specimen was then bolted into the joint testing machine with a stifle angle of 135°. The flexion/extension angle of the stifle could be changed during testing as the joint testing machine permitted the movement of the femur in increments of 5°. Cranial/caudal translation of the distal tibia during loading was prevented by passing a 2.0 mm diameter strand of nylon, attached to the joint testing machine cranial and caudal to the specimen, through a bone tunnel drilled from medial to lateral in the distal tibia. The angle of the stifle was confirmed with a manual goniometer prior to testing and the specimen was periodically sprayed with saline which ensured that it was kept moist throughout the period of preparation and testing.

**Figure 1:**
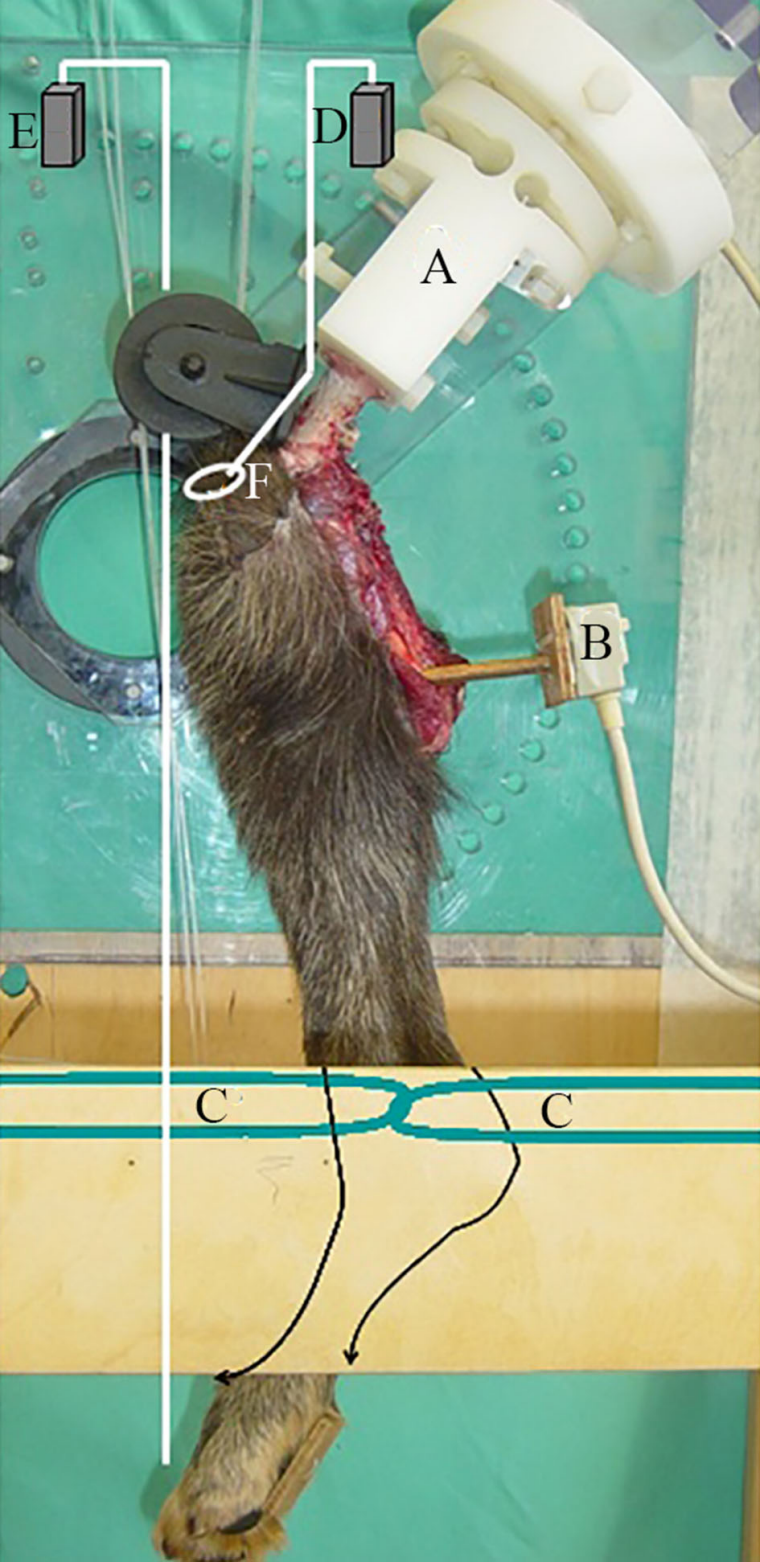
Schematic representation of the loads applied to the patella and pes via a system of weights and pulleys within the joint testing machine. The position of the sensor and the restraint of the distal tibia are also illustrated. **(A)** Bone clamp, **(B)** Sensor, **(C)** Restraint of distal tibia, **(D)** Quadriceps load, **(E)** Pes load, (e) Patella.

The specimen was loaded *via* a system of weights and pulleys, which are schematically represented in Figure 1. The ground reaction force was generated via a vertical load applied to the metatarsal pad, and the force in the quadriceps muscle was simulated via a load applied to the patella in the general direction of action of the quadriceps muscle.

### Points of Interest and Definition of Axes

**Figure 2:**
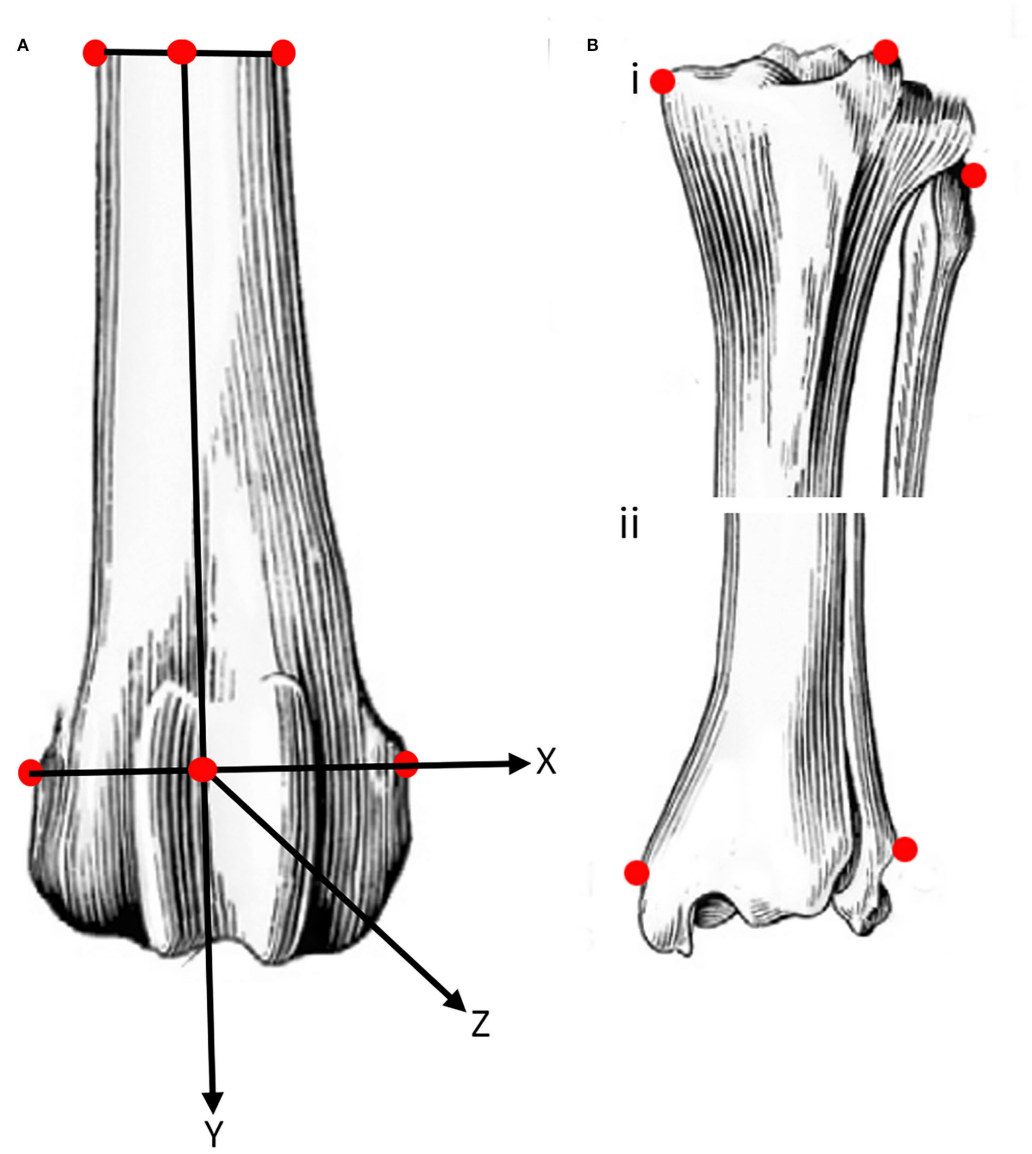
Schematic representation of the location (see text for details) of points of interest (POI) on easily identifiable prominent landmarks on the femur **(A)**, and proximal **(B**^**i**^**)** and distal **(B**^**ii**^**)** tibia and fibula. The POI of the femur were used to define a system of axes embedded in the femur with the origin midway between the points of interest on the distal femur. The POI on the caudal aspect of the tibia at the attachment of the sensor is not shown.

Coordinates of 10 points of interest (POI), were collected from the tibia, fibula and femur at the beginning of testing and each time the angle of the stifle was changed. The POI on the femur (Figure 2) were the lateral and medial epicondyles, distally, and the lateral and medial cortex distal to the PMMA, proximally. The three POI on the proximal tibia and fibula were the fibula head, medial tibial condyle, and the tibial tuberosity, and the two distal points were the medial and lateral maleoli. The final point on the tibia was selected at the attachment of the sensor to the caudal tibial cortex.

The four femoral POI were used to define a system of axes embedded in the femur. The origin of the system of axes was defined as the center of the line connecting the two distal femoral points. Similarly, the center of the line connecting the two proximal points of the femur was determined. The z- axis was defined as a line connecting the origin and the center of the line between the proximal femoral points. A plane was then defined using the two center points, previously defined, and the point on the medial femoral epicondyle. The y- axis was defined as a line perpendicular to the defined plane and passing through the origin. The x- axis was defined as a line perpendicular to the two previously defined axes and passing through the origin (Figure 2).

### Specimen Testing

All specimens were initially tested with the CCL intact (CCLI). A single test comprised the loading of the metatarsal pad, in a vertical to the ground, with 10 weights, 0.5, 1.0, 1.5, 2.0, 2.5, 3.0, 3.5, 4.0, 4.5, and 5.0 kg. Each specimen was tested with the stifle fully extended (135°), at 15° of flexion, and 30° of flexion, and at each angle, the specimen was tested with quadriceps loads of 0, 5.0, and 10.0 kg.

**Figure 3:**
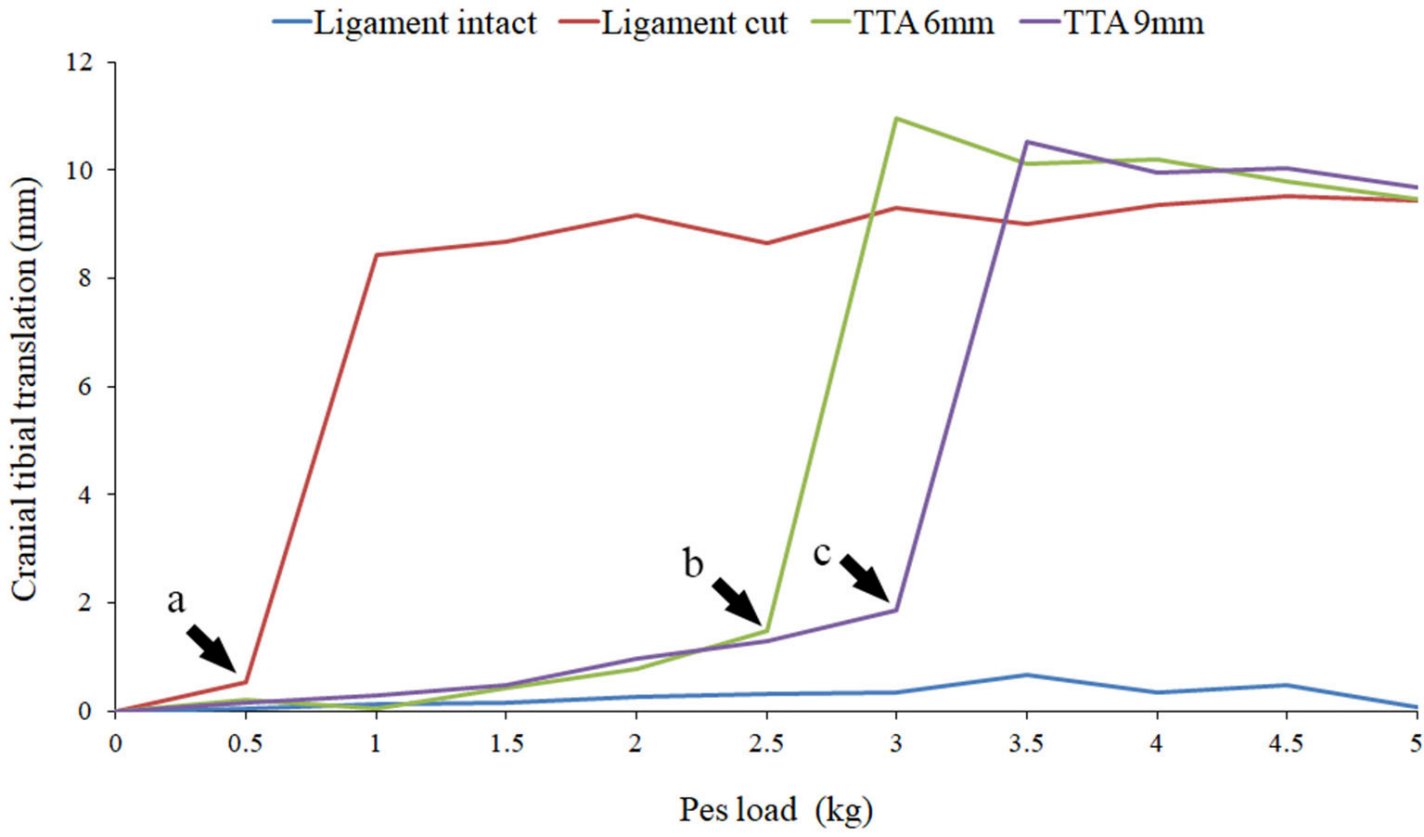
The results of Specimen 5 with the stifle joint at 120° of flexion and a quadriceps load of 5 kg are illustrated in this graph. Results with the cruciate ligament intact (light blue), cut (dark red), TTA 6 mm (light green) and TTA 9 mm (dark green) at all the pes loads are represented. The loads prior to failure are marked by the arrow heads. At pes loads exceeding the point of failure a marked increase in cranial translation of the tibia was noted.

Once this was completed the specimen was removed from the joint testing machine and the stifle joint was approached via a mini medial arthrotomy. The CCL was exposed and sectioned, and the joint capsule, medial retinaculum, subcutaneous tissue, and skin were closed with a simple continuous suture. The specimen was then re-tested with CCL cut (CCLC) using the identical protocol. Subsequent to this the specimen was removed from the joint testing machine and a TTA was performed using the described technique (1). The specimen was then tested for failure with both a 6 mm cage (CCLTTA6) and a 9 mm cage (CCLTTA9). Failure of the repair was identified by a sudden increase in translation of the tibia post-TTA which was similar in magnitude to the tibial translation in the CCLC specimens (prior to repair). Failure was seen during testing as an abrupt, marked cranial translation of the tibia as the load to the pes was applied (Figure 3). The largest load applied to the pes prior to the abrupt increase in cranial tibial translation was identified by plotting the tibial translation of all four tests on a single set of axes.

### Data Processing

The output of the NOB consisted of the x, y, and z coordinates of the sensor in the coordinate system generated by the transmitter, as well as a 3 × 3 rotational matrix resulting in a row of data with 12 values. For each load, the cranial translation of the tibia was calculated by determining the difference in the position of the loaded tibia relative to the unloaded tibia. The position of the tibia was initially measured 10 times with the pes unloaded. A load was then applied to the pes and the position under load was measured 10 times. This was repeated for all loads resulting in 210 measurements per test. For specimen 1, 21, 210 × 12 matrices were generated while for specimens 2–6, 36 210 × 12 matrices were generated for each specimen. Data from 13 tests were lost due to a storage malfunction.

The initial step was to define a system of axes embedded in the femur based on the POI as previously described. Once the system of axes embedded in the femur was defined, the location of its origin was determined in the global coordinate system and the two systems of axes were aligned. This enabled us to describe the translation and rotation of the sensor connected to the tibia relative to the system of axes embedded in the femur.

A similar manipulation was performed in order to describe the movement of the sensor, attached to the tibia, and the POI which coincided with the insertion of the patella tendon on the tibia was chosen to characterize the translation of the tibia relative to the femur. All data manipulation was performed using MATLAB 7.0 (The Mathworks, INC; Natick, MA United States) software with custom-written software.

## Results

### Ligament Intact

Cranial translation of the tibia relative to the femur in the CCLI specimens increased with increasing loads applied to the pes. This is illustrated in Figure 3 which is the data obtained from specimen 5 at a stifle joint angle of 120° and with a quadriceps load of 5.0 kg. Similar results were obtained for all joint angles and all quadriceps loads, for all specimens. The mean cranial translation under 5.0 kg of pes load was calculated using all available data. With the stifle joint at an angle of 135° the tibia translated 4.09 ± 3.35 mm (mean ± sd), 3.90 ± 4.61 mm (mean ± sd), and 5.25 ± 4.11 mm (mean ± sd) with quadriceps loads of 0, 5, and 10 kg, respectively. With the stifle joint at an angle of 120° and under identical loading conditions the tibia translated 3.51 ± 2.87 mm (mean ±sd), 1.63 ± 1.19 mm (mean ±sd), and 3.21 ± 4.09 mm (mean ±sd) with quadriceps loads of 0, 5, and 10 kg, respectively. Similarly, with the joint at an angle of 105° the tibia translated 2.75 ± 2.14 mm (mean ±sd), 2.24 ± 2.10 mm (mean ±sd), and 2.92 ± 2.16 mm (mean ±sd) with quadriceps loads of 0, 5, and 10 kg, respectively.

### Ligament Cut

Cranial translation of the tibia relative to the femur, in the CCLC specimens was similar in all specimens and was characterized by marked cranial translation under the lowest loads, followed by a plateau where increasing loads applied to the pes resulted in only a minor increase in tibial translation (Figure 3). The mean cranial translation, under 5.0 kg of pes load, was calculated using all available data. With the stifle joint at an angle of 135° the tibia translated 7.32 ± 2.59 mm (mean ±sd), 11.21 ± 3.80 mm (mean ±sd), and 12.27 ± 4.57 mm (mean ±sd) with quadriceps loads of 0, 5 and 10 kg, respectively. With the stifle joint at an angle of 120° and under identical loading conditions the tibia translated 11.93 ± 8.22 mm (mean ± sd), 13.45 ± 8.14 mm (mean ± sd), and 12.03 ± 6.95 mm (mean ± sd) with quadriceps loads of 0, 5, and 10 kg, respectively. Similarly, with the joint at an angle of 105° the tibia translated 10.49 ± 6.61 mm (mean ±sd), 11.75 ± 6.27 mm (mean ± sd), and 12.39 ± 5.83 mm (mean ± sd) with quadriceps loads of 0, 5, and 10 kg, respectively.

### TTA 6 mm and TTA 9 mm

Tibial tuberosity advancements of 6 and 9mm were tested with no quadriceps load, however, this data was not included as the translation of the tibia under these loading conditions was identical to the tibial translation seen immediately after cutting the CCL. Data from 64 tests performed on TTA 6 mm and TTA 9 mm specimens, at all three flexion angles with the quadriceps loaded with 5 and 10 kg were available for analysis (Tables 1A–C). In 58/64 tests done after TTA, there was a minimal translation of the tibia relative to the femur until with increasing load applied to the pes, however, above a load that differed between specimens, maximum translation of the tibia relative to the femur was seen with minimal increase in translation with further increase in load (Figure 3; Table 3). In these tests, the pes load prior to failure could be identified. The absolute translations (mm) of the tibia relative to the femur at the load prior to failure (Table 1), are listed in Table 2. In 38/62 tests the tibia translated cranially <2 mm. In 18/62 tests the tibia translated between 2 and 4 mm and in six tests the tibia translated between 4 and 5 mm, cranially.

**Table 1A:** The change in the pes load prior to failure with increasing quadriceps load applied to the patella.

		**Specimen 1**		**Specimen 2**		**Specimen 3**		**Specimen 4**		**Specimen 5**		**Specimen 6**	
		**TTA**	**TTA**	**TTA**	**TTA**	**TTA**	**TTA**	**TTA**	**TTA**	**TTA**	**TTA**	**TTA**	**TTA**
		**6 mm**	**9 mm**	**6 mm**	**9 mm**	**6 mm**	**9 mm**	**6 mm**	**9 mm**	**6 mm**	**9 mm**	**6 mm**	**9 mm**
Stifle angle 135°	5 kg quad load	2.0 kg		1.5 kg	1.0 kg	0.5 kg	5.0 kg	0.5 kg	1.0 kg			0.5 kg	1.0 kg
	10 kg quad load	3.5 kg		1.5 kg	1.5 kg	1.5 kg	5.0 kg	1.5 kg	1.5 kg			1.5 kg	2.0 kg
Stifle angle 120°	5 kg quad load	2.5 kg		0.5 kg	2.0 kg	1.5 kg	2.5 kg	2.0 kg	2.0 kg	2.5 kg	3.0 kg	1.0 kg	1.5 kg
	10 kg quad load	3.0 kg		1.0 kg	2.5 kg	2.0 kg	3.0 kg	2.0 kg	2.0 kg	3.0 kg	4.0 kg	1.5 kg	2.0 kg
Stifle angle 105°	5 kg quad load	1.0 kg		0.5 kg	2.0 kg	2.0 kg	5.0 kg	0.5 kg	1.0 kg	1.5 kg	1.5 kg	0.5 kg	1.0 kg
	10 kg quad load	2.0 kg		1.0 kg	3.5 kg	2.5 kg	5.0 kg	1.5 kg	2.0 kg	2.5 kg	3.0 kg	1.0 kg	2.0 kg

*Color added for emphasis*.

**Table 1B:** The change in the pes load prior to failure with increasing tibial tuberosity advancement.

		**Specimen 1**		**Specimen 2**		**Specimen 3**		**Specimen 4**		**Specimen 5**		**Specimen 6**	
		**TTA**	**TTA**	**TTA**	**TTA**	**TTA**	**TTA**	**TTA**	**TTA**	**TTA**	**TTA**	**TTA**	**TTA**
		**6 mm**	**9 mm**	**6 mm**	**9 mm**	**6 mm**	**9 mm**	**6 mm**	**9 mm**	**6 mm**	**9 mm**	**6 mm**	**9 mm**
Stifle angle 135°	5 kg quad load	2.0 kg		1.5 kg	1.0 kg	0.5 kg	5.0 kg	0.5 kg	1.0 kg			0.5 kg	1.0 kg
	10 kg quad load	3.5 kg		1.5 kg	1.5 kg	1.5 kg	5.0 kg	1.5 kg	1.5 kg			1.5 kg	2.0 kg
Stifle angle 120°	5 kg quad load	2.5 kg		0.5 kg	2.0 kg	1.5 kg	2.5 kg	2.0 kg	2.0 kg	2.5 kg	3.0 kg	1.0 kg	1.5 kg
	10 kg quad load	3.0 kg		1.0 kg	2.5 kg	2.0 kg	3.0 kg	2.0 kg	2.0 kg	3.0 kg	4.0 kg	1.5 kg	2.0 kg
Stifle angle 105°	5 kg quad load	1.0 kg		0.5 kg	2.0 kg	2.0 kg	5.0 kg	0.5 kg	1.0 kg	1.5 kg	1.5 kg	0.5 kg	1.0 kg
	10 kg quad load	2.0 kg		1.0 kg	3.5 kg	2.5 kg	5.0 kg	1.5 kg	2.0 kg	2.5 kg	3.0 kg	1.0 kg	2.0 kg

*Color added for emphasis*.

**Table 1C:** The change in the pes load prior to failure with increasing tibial tuberosity advancement and quadriceps load.

		**Specimen 1**		**Specimen 2**		**Specimen 3**		**Specimen 4**		**Specimen 5**		**Specimen 6**	
		**TTA**	**TTA**	**TTA**	**TTA**	**TTA**	**TTA**	**TTA**	**TTA**	**TTA**	**TTA**	**TTA**	**TTA**
		**6 mm**	**9 mm**	**6 mm**	**9 mm**	**6 mm**	**9 mm**	**6 mm**	**9 mm**	**6 mm**	**9 mm**	**6 mm**	**9 mm**
Stifle angle 135°	5 kg quad load	2.0 kg		1.5 kg	1.0 kg	0.5 kg	5.0 kg	0.5 kg	1.0 kg			0.5 kg	1.0 kg
	10 kg quad load	3.5 kg		1.5 kg	1.5 kg	1.5 kg	5.0 kg	1.5 kg	1.5 kg			1.5 kg	2.0 kg
Stifle angle 120°	5 kg quad load	2.5 kg		0.5 kg	2.0 kg	1.5 kg	2.5 kg	2.0 kg	2.0 kg	2.5 kg	3.0 kg	1.0 kg	1.5 kg
	10 kg quad load	3.0 kg		1.0 kg	2.5 kg	2.0 kg	3.0 kg	2.0 kg	2.0 kg	3.0 kg	4.0 kg	1.5 kg	2.0 kg
Stifle angle 105°	5 kg quad load	1.0 kg		0.5 kg	2.0 kg	2.0 kg	5.0 kg	0.5 kg	1.0 kg	1.5 kg	1.5 kg	0.5 kg	1.0 kg
	10 kg quad load	2.0 kg		1.0 kg	3.5 kg	2.5 kg	5.0 kg	1.5 kg	2.0 kg	2.5 kg	3.0 kg	1.0 kg	2.0 kg

*Color added for emphasis*.

**Table 2:** Absolute translation (mm) of the tibia relative to the femur at the load prior to failure listed in Table 1.

		**Specimen 1**		**Specimen 2**		**Specimen 3**		**Specimen 4**		**Specimen 5**		**Specimen 6**	
		**TTA**	**TTA**	**TTA**	**TTA**	**TTA**	**TTA**	**TTA**	**TTA**	**TTA**	**TTA**	**TTA**	**TTA**
		**6 mm**	**9 mm**	**6 mm**	**9 mm**	**6 mm**	**9 mm**	**6 mm**	**9 mm**	**6 mm**	**9 mm**	**6 mm**	**9 mm**
Stifle angle 135°	5 kg quad load	3.25		3.89	2.32	0.79	4.93	0.52	3.59			0.14	1.66
	10 kg quad load	2.69		4.04	0.21	1.01	4.18	1.33	1.95			0.51	1.46
Stifle angle 120°	5 kg quad load	4.17		0.37	1.63	0.67	1.01	3.34	0.70	1.48	1.87	1.64	1.24
	10 kg quad load	3.45		0.71	1.76	1.11	1.00	2.40	0.55	1.57	2.17	1.32	1.42
Stifle angle 105°	5 kg quad load	3.73		0.52	3.77	2.29	1.50	0.02	1.37	1.60	2.37	1.26	2.16
	10 kg quad load	3.87		0.87	3.78	0.60	1.32	0.30	2.25	2.40	1.73	2.77	4.12

**Table 3:** Translation of the tibia subsequent to performing the TTA relative to the translation of the tibia with the ligament cut at the loads listed in Table 1.

		**Specimen 1**		**Specimen 2**		**Specimen 3**		**Specimen 4**		**Specimen 5**		**Specimen 6**	
		**TTA**	**TTA**	**TTA**	**TTA**	**TTA**	**TTA**	**TTA**	**TTA**	**TTA**	**TTA**	**TTA**	**TTA**
		**6 mm**	**9 mm**	**6 mm**	**9 mm**	**6 mm**	**9 mm**	**6 mm**	**9 mm**	**6 mm**	**9 mm**	**6 mm**	**9 mm**
Extended	5 kg quad load	0.52		0.95	0.35	3.53	0.64	0.22	0.78			0.12	0.20
	10 kg quad load	0.25		0.42	0.12	0.35	0.74	0.20	0.29			0.04	0.12
15° Flex	5 kg quad load	0.30		0.44	0.49	0.73	0.22	0.50	0.10	0.17	0.17	0.88	0.06
	10 kg quad load	0.22		0.39	0.38	0.35	0.22	0.38	0.09	0.17	0.23	0.07	0.07
30° Flex	5 kg quad load	0.59		0.20	0.29	0.57	0.24	0.02	0.39	0.28	0.42	1.53	0.22
	10 kg quad load	0.33		0.17	0.24	0.13	0.20	0.05	0.38	0.28	0.19	0.45	0.35

TTA failed to decrease cranial tibial translation in 6 tests which were characterized by a marked tibial translation under the smallest (0.5 kg) pes load. Load to failure was considered to be 0.5 kg in these tests, and all occurred with a 6 mm cage and a quadriceps load of 5 kg. When these specimens were retested with a 9 mm cage and/or a quadriceps load of 10 kg, the initial phase of decreased translation was seen, and a load prior to failure could be identified. Two of these failures occurred with the stifle at 135°, one occurred with the stifle joint at 120° and three occurred with the stifle joint at 105°. The loads prior to failure with increasing TTA(joint angle and quadriceps load constant) are listed in Table 1A. Twenty-eight pairs of tests were available for comparison. The pes load prior to failure increased when the tibial tuberosity was advanced from 6 to 9 mm in 22/28 pairs of tests. In five tests, the load prior to failure stayed the same, and in one test the load prior to failure decreased.

The loads prior to failure with increasing quadriceps load (joint angle and TTA constant) are listed in Table 1B. Thirty-one pairs of tests (17 tests with the 6 mm cage and 14 tests with the 9 mm cage) were available for comparison. The load prior to failure increased when the quadriceps load was increased from 5.0 to 10.0 kg in 15/17 tests with a 6 mm cage and in 11/14 tests with a 9 mm cage. In tests in which the load to failure was not reached (Specimen 3 at stifle angles of 135° and 105° and Specimen 4 at a stifle angle of 120°) a load of 5.0 kg was recorded). The loads prior to failure with increasing quadriceps load and TTA(joint angle constant) are illustrated in Table 1C. The load prior to failure increased with a combination of increased cage width and quadriceps load in 10/11 tests with only 1 exception (Specimen 4 at a stifle angle of 120°).

## Discussion

TTAhas been shown to be clinically successful in dogs with rupture of the CCL and has been reported to result in a rapid return to weight bearing and high levels of function (1). However, subluxation of the stifle demonstrated on weight-bearing horizontal-beam radiographs was shown to persist in 21 of 30 dogs treated with TTA for 18 ± 14 months (mean ± SD) postoperatively (13). The finding of persistent stifle instability at 8–12 weeks postoperatively in dogs treated with TTA has also been shown using *in-vivo* using uniplanar fluoroscopic kinematography (14). *In-vivo* instability is in contrast to the findings of biomechanical studies which have shown that TTA is effective in decreasing the cranial translation of the tibia relative to the femur in the cranial cruciate ligament deficient stifle. In one biomechanical study that cranial tibial translation was decreased but not entirely eliminated after performing a TTA (12). However, in a subsequent study (15) advancing the tibial tuberosity resulted in an almost linear decrease in all the measured loads with sufficient advancement resulting in the elimination of cranial tibial thrust. In a further study, it was shown that TTA not only neutralizes cranial subluxation of the tibia but caused a caudal shift in the tibia relative to the femur (11).

The marked stifle instability is seen subsequent to performing TTA in cranial cruciate ligament deficient stifle joints (13, 14) was demonstrated in this study, however, the use of cadaveric limbs and cutting of several muscles insert on the tibia, may have exaggerated our findings. We loaded the pes in an effort to simulate normal weight bearing, and while it is unlikely that *in-vitro* loading is identical to *in-vivo* loading, marked instability was seen. Based on the theoretical considerations underlying the TTA procedure, increasing tibial tuberosity advancement, quadriceps load, and stifle flexion angle was expected to increase the caudally directed tibio-femoral shear force. The effect of each of these factors was tested, over a range of joint angles, by incrementally increasing the load on the pes, which increased the cranially directed tibio-femoral shear force generated by tibial compression. Under these testing conditions, the cranial cruciate deficient stifles treated with TTA were initially stable but became unstable in 60/64 tests under higher loads. Increasing the advancement of the tibial tuberosity and increasing the quadriceps load was effective in counteracting the cranially directed tibio-femoral shear force and may provide a basis for the development of strategies to improve stability in clinical cases.

Increased TTA and quadriceps loads were shown to act independently in maintaining the stability of a cranial cruciate ligament deficient stifle joint. Increasing either the amount of TTA or the quadriceps load increased the load which could be resisted by the repair, with the combination of both being the most effective. The load prior to failure increased, in 22/28 pairs of tests, when the TTA was increased from 6 to 9 mm and is explained by consideration of the quadriceps lever arm. The distance from the femorotibial contact point to the insertion of the patella tendon is considered to be the lever arm of the quadriceps muscle which increases up to 10% when performing a TTA (16). For each quadriceps load, replacing the 6 mm cage with a 9 mm cage increases the lever arm of the quadriceps muscle, which results in a larger moment, which would be more effective in limiting cranial translation. The force generated by the quadriceps muscle is the source of the caudally directed tibio-femoral shear force. It can be expected that the greater the quadriceps load, the greater the caudally directed horizontal component. Increasing quadriceps load resulted in an increase in the load prior to failure in 28/31 tests. The combination of increasing cage width and quadriceps load only failed to increase the load prior to failure in 1 specimen; however, in this specimen, the load prior to failure exceeded the maximum load of 2 kg under all testing conditions.

It was expected that decreasing PTA by flexing the stifle would increase the load to failure due to an increase in the caudally directed tibio-femoral shear force, however, in this study 60° of joint flexion did not increase the ability of TTA to resist a larger cranially directed tibio-femoral shear force. With increasing joint flexion, load prior to failure only increased in 3/18 tests available for comparison. This was surprising as in this experimental setup increasing tibial tuberosity translation and increasing quadriceps load both affected the biomechanics of the stifle as expected. TTAis based on a static model and the predicted behavior of this model at stifle angles other than the angle on which the model is based can be questioned. Angles examined in this study were limited to reflect the stance phase of the stride, and it is possible that the effect of flexion angle only becomes significant at angles greater than the angles seen during locomotion. The relationship between the stifle angle and the caudally directed tibio-femoral shear force remains unclear.

One potential limitation of this study is that TPA was not measured after performing TTA. This study was not designed to evaluate the TTA technique, but rather to show the effects of increasing tibial tuberosity advancement, quadriceps load, and decreasing stifle angle on the biomechanics of the stifle joint. It was clearly demonstrated that under these testing conditions the stability provided by the different elements could be overcome by increasing the load applied to the foot, and failure was characterized by sudden marked cranial translation of the tibia relative to the femur. We suspect that instability seen in clinical cases treated with TTA has the same explanation. A further limitation is that the loads applied were not necessarily equivalent to physiological loads. Quadriceps loads that were applied in this experiment were up to 50% of BW and pes loads that were applied were up to 25% of body weight. This was due mainly to the limitations of the testing system. Biomechanical analysis has shown that the quadriceps force may be as much as 95% of BW at 80% of the stance phase (17). In our experimental setup this would have required quadriceps loads in excess of 20 kg which exceeded the limitations of the joint testing machine. It is likely that, in this study, the loads applied as well as the ratio between the pes load and the quadriceps load were not representative of physiological loading conditions.

## Data Availability Statement

The original contributions presented in the study are included in the article/supplementary material, further inquiries can be directed to the corresponding author.

## Ethics Statement

The animal study was approved by Koret School of Veterinary Medicine Institutional Animal Care and Use Committee, Hebrew University of Jerusalem, Israel (Approval Number: KSVM-VTH/31_2019).

## Author Contributions

CO, JM, and CB contributed to the conception and design of the study, data collection, and interpretation. JM, CB, and LC wrote the first draft and major revisions of the paper. All authors contributed to manuscript revision and read and approved the submitted version.

## Funding

The publication was supported by funds from the National Research Development Projects to Finance Excellence (PFE)-14 (ID 546) granted by the Romanian Ministry of Research, Innovation and Digitalization.

## Conflict of Interest

The authors declare that the research was conducted in the absence of any commercial or financial relationships that could be construed as a potential conflict of interest.

## Publisher's Note

All claims expressed in this article are solely those of the authors and do not necessarily represent those of their affiliated organizations, or those of the publisher, the editors and the reviewers. Any product that may be evaluated in this article, or claim that may be made by its manufacturer, is not guaranteed or endorsed by the publisher.
